# Nanopore Discrimination of Coagulation Biomarker Derivatives and Characterization of a Post-Translational Modification

**DOI:** 10.1021/acscentsci.2c01256

**Published:** 2023-02-03

**Authors:** Aïcha Stierlen, Sandra J. Greive, Laurent Bacri, Philippe Manivet, Benjamin Cressiot, Juan Pelta

**Affiliations:** † LAMBE, CNRS, CY Cergy Paris Université, 95033 Cergy, France; ‡ DreamPore S.A.S., 33 Boulevard du Port, 95000 Cergy, France; § LAMBE, CNRS, Univ Evry, Université Paris-Saclay, 91025 Evry-Courcouronnes, France; ∥ Centre de Ressources Biologiques Biobank Lariboisière (BB-0033-00064), DMU BioGem, AP-HP, 75475 Paris, France; ⊥ Université Paris Cité, Inserm, NeuroDiderot, F-75019 Paris, France

## Abstract

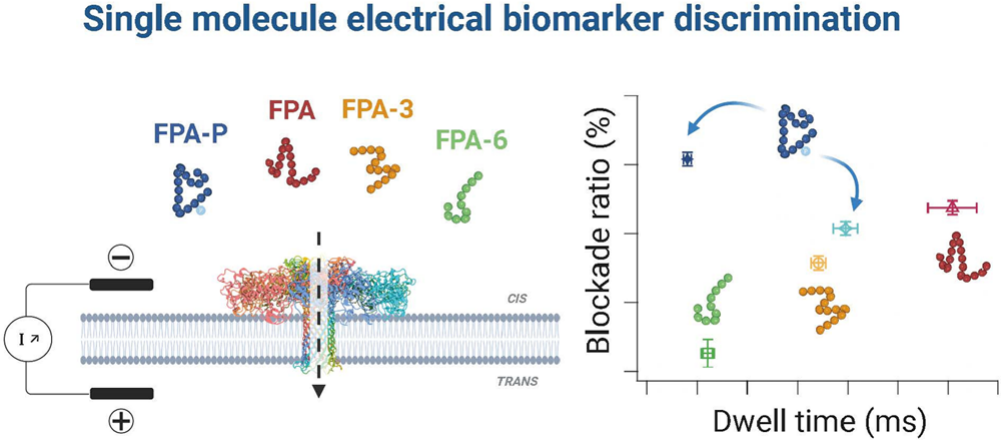

One of the most important health challenges is the early and ongoing detection of disease for prevention, as well as personalized treatment management. Development of new sensitive analytical point-of-care tests are, therefore, necessary for direct biomarker detection from biofluids as critical tools to address the healthcare needs of an aging global population. Coagulation disorders associated with stroke, heart attack, or cancer are defined by an increased level of the fibrinopeptide A (FPA) biomarker, among others. This biomarker exists in more than one form: it can be post-translationally modified with a phosphate and also cleaved to form shorter peptides. Current assays are long and have difficulties in discriminating between these derivatives; hence, this is an underutilized biomarker for routine clinical practice. We use nanopore sensing to identify FPA, the phosphorylated FPA, and two derivatives. Each of these peptides is characterized by unique electrical signals for both dwell time and blockade level. We also show that the phosphorylated form of FPA can adopt two different conformations, each of which have different values for each electrical parameter. We were able to use these parameters to discriminate these peptides from a mix, thereby opening the way for the potential development of new point-of-care tests.

## Abstract 2

Aerolysin nanopore sensing for discrimination between fibrinopeptide A (FPA) post-translationally modified (phosphorylated; FPA-P) and proteolytically cleaved forms (FPA3 and FPA6).

## Introduction

**Figure 1:**
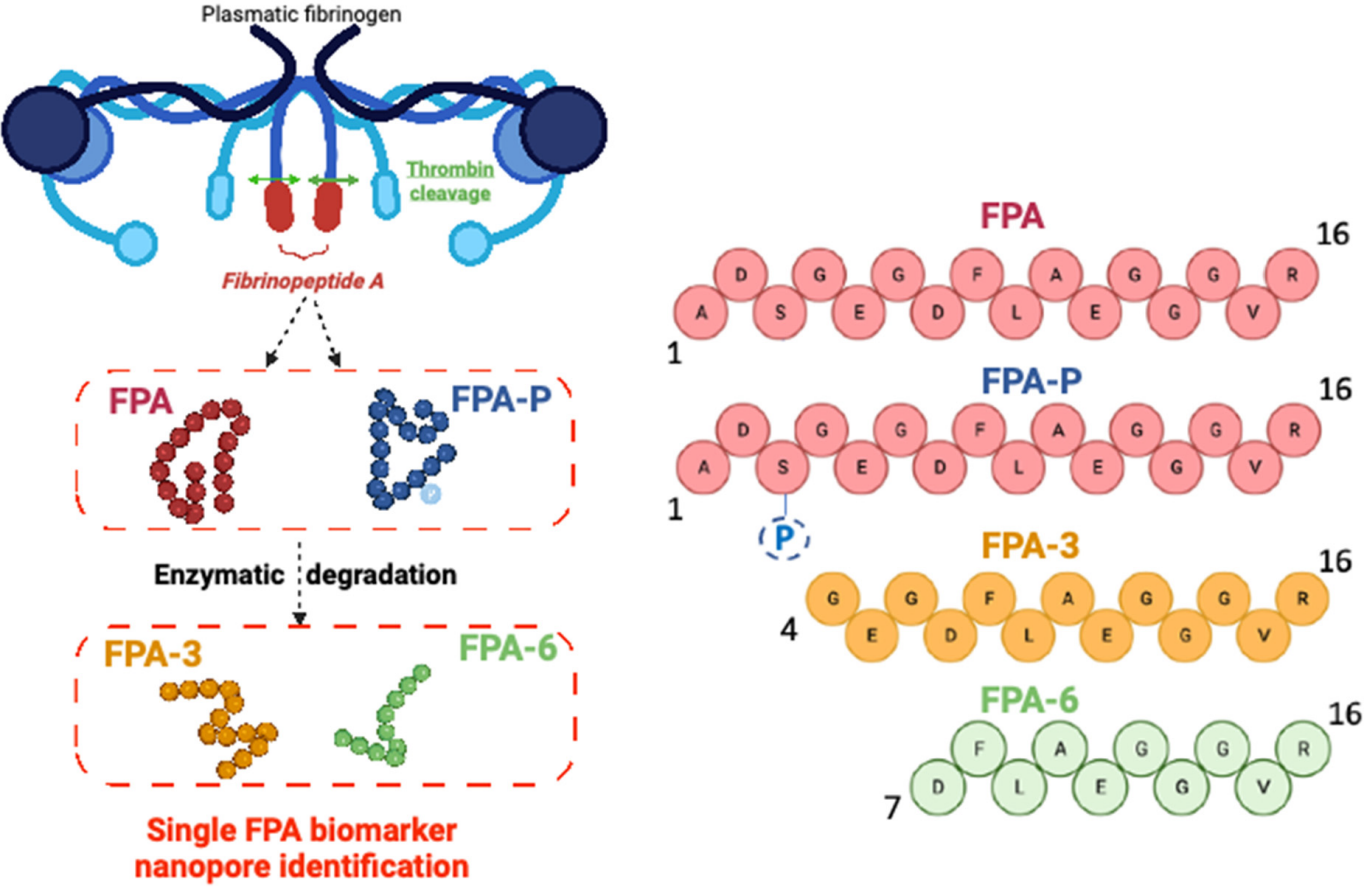
The fibrinopeptide A (FPA) family of biomarkers. Cartoon depiction (top left image) of the cleavage of the 16 amino acid N-terminal peptide (red oblong) from fibrinogen (represented by the blue lines and shapes) by thrombin (green arrows) to release FPA during thrombogenesis. FPA exists in several forms in blood, unphosphorylated (red) and phosphorylated (blue), and as a series of sequential N-terminal amino acid cleavage products produced by enzymatic degradation (FPA1–6), with FPA3 (yellow) and FPA6 (green) shown above. Illustrations created with BioRender.com.

Coagulation (blood clotting) disorders, such as thrombosis in venous thromboemulism (VTE) and disseminated intravascular coagulation^1^ (DIC), play a role in many cancers, ischemic heart disease, stroke, systemic lupus erythematosus, and COVID.^2−7^ Fibrinopeptide A (FPA) is a clinical biomarker for coagulation, one of several used to diagnose and manage treatment for clotting disorders. The clotting process is tightly regulated by the coagulation cascade, a complex multiprotein network of regulatory and feedback loops. A key event in this process is the release of FPA from the N-termini of the α-chains of the fibrinogen dimer by thrombin cleavage—hence, its utility as a biomarker for thrombogenesis (Figure 1). Subsequent thrombin cleavage events on the β and γ chains allow the fibrin monomers to polymerize into a network of fibers to form a clot.^8^ This process is normally tightly regulated and evenly balanced by fibrinolysis, the clot lysis process, which is mediated by plasmin.^9^ Dysregulation of this balance underlies many clotting and hemorrhagic disorders.^8,9^

The 16 amino acid FPA is not only found in blood, but is also excreted in urine, with levels increasing in both after heart attack.^10,11^ In normal adult serum and plasma samples, it exists in two forms: unphosphorylated, and phosphorylated at Ser3 (FPA-P, ∼20% of total FPA)^12^ (Figure 1). A similar level of relative phosphorylation (at both Ser3 and Ser345) was observed for both blood-derived and recombinantly expressed fibrinogen.^13,14^ The proportion of FPA-P in serum or plasma increases after surgery and heart attack, during VTE and DIC events, and in ovarian cancer,^15−20^ which suggests that phosphorylation level plays a role in regulating thrombosis. Indeed, complementary biochemical assays showed that the phosphorylation of fibrinogen reduced clotting times,^21^ likely because of the increased affinity of the phosphorylated N-terminus (phosphoSer3) for the thrombin active site compared with the unphosphorylated protein.^22^ This effectively increases the apparent rate of FPA-P release at lower concentrations of thrombin compared with FPA.^23^ Phosphorylation also increased clot resistance to fibrinolysis by plasmin.^24^

Despite the potential utility of FPA-P in the diagnosis and management of thrombosis, VTE, and DIC, current clinical assays for FPA are long, complicated antibody-based approaches that are hampered by cross-reactivity with FPA-P and fibrinogen,^2^ as are assays for other biomarkers of thrombogenesis and fibrinolysis.^8^ These assays are further complicated by ongoing proteolysis in serum and plasma samples.^25−27^ By mass spectrometry, a series of 6 FPA derivative peptides (FPA1–6) have been identified (FPA-3 and FPA-6 shown in Figure 1) where the N-terminal amino acid has sequentially been cleaved.^26−28^ Through the use of this method, these peptides have been found in higher concentration in samples from cancer patients than in normal samples.^28,29^ However, the efficacy of mass spectrometry as routine clinical assays for cancer remains to be proven. A rapid, high-throughput, low-cost, high-resolution assay that can discriminate between and quantify relative levels of FPA and FPA-P and its derivative peptides would allow an accurate determination of FPA levels and facilitate the collection of large data sets to correlate relative levels of FPA/FPA-P with disease pathology. This data would also contribute to the understanding of the role of fibrinogen phosphorylation in regulating thrombogenesis and fibrinolysis. Further adaptation to a point-of-care (POC) assay could vastly improve the cost and efficiency of treatment delivery for diseases related to thrombosis, VTE, and DIC.

While there is a clear need to develop novel sensitive and low-cost medical analysis tools for diagnostics purposes, many currently available POC tests rely on antibody-based detection, which for some biomarkers (eg FPA), can have reduced specificity because of cross-reactivity. Nanopore detection has offered an alternative high-resolution detection method with proven on-site utility during recent pandemics. Indeed, this method allowed the identification of variations in the sequences of the ebola^30^ and SARS-CoV-2 viruses.^31−34^ However, the nanopore detection of peptides and proteins is a more recent development with only a few studies up to now that have used nanopores to directly analyze peptide or protein biomarkers,^35−38^ or to indirectly detect these molecules.^39−44^ This method can also be used to identify chemical or post-translational modifications of peptides^45−51^ or proteins.^52^

**Figure 2:**
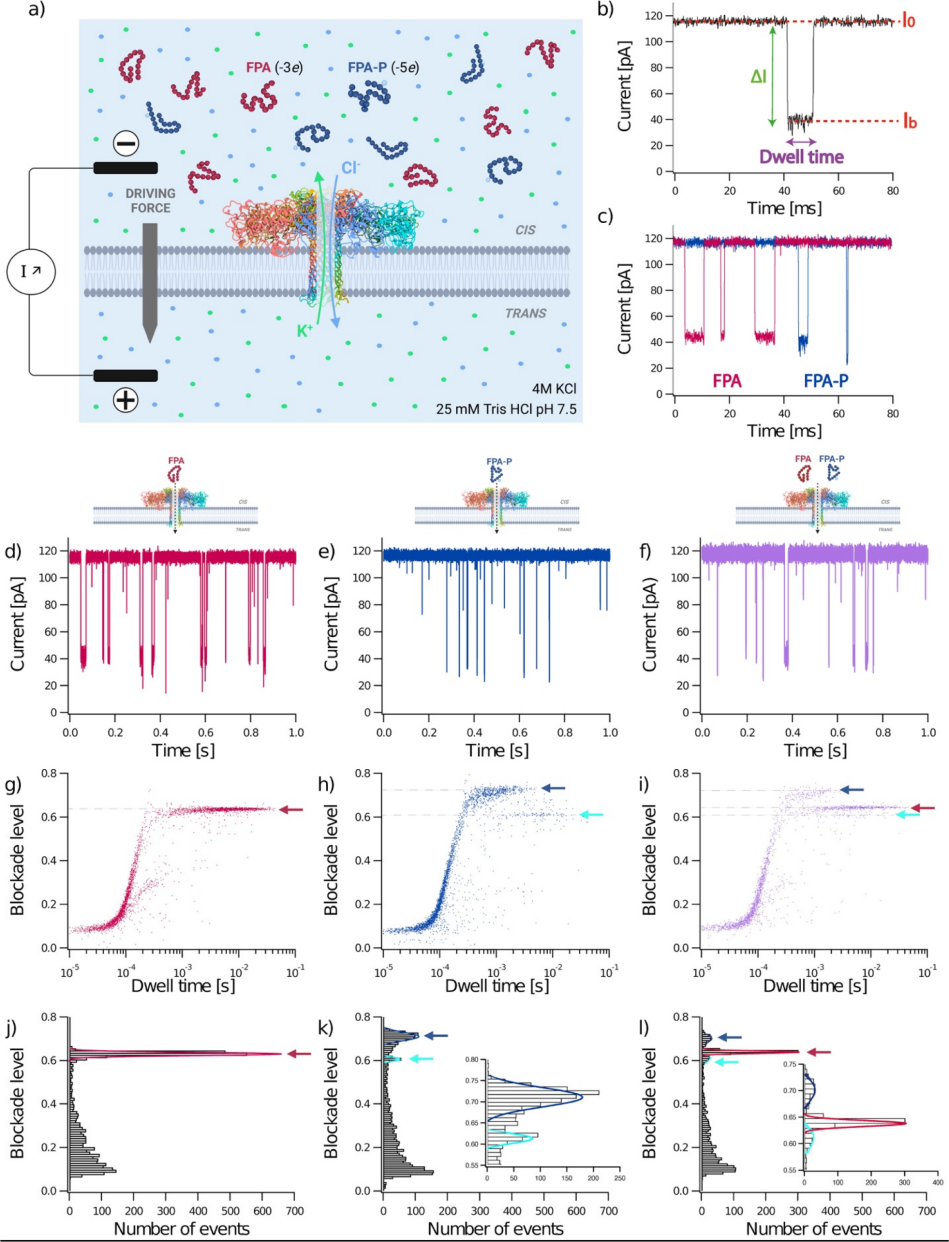
Characterization and discrimination of a post-translational modification in a peptide biomarker. A depiction of the experimental setup (a) where FPA (red) and FPA-P (blue) peptides were analyzed separately at 40 μM final concentration (d,e,g,h) and together in an equimolar mixture [10 μM; purple data in (f,i), using a wild-type aerolysin nanopore (ribbon depiction of PDB: 5JZT)^68^ inserted in a lipid membrane. When a voltage is applied, K^+^ and Cl^–^ ions (pale green and pale blue spheres) in the buffer flow through the pore (pale green and pale blue arrows], thereby resulting in an ionic current (*I*_0_; b). FPA peptides have a net negative charge of −3e and −5e for FPA and FPA-P, respectively (a). A typical current trace recorded over 80 ms (b), showing the open pore current (*I*_0_) and blockade current (*I*_b_), blockade depth (Δ*I*), and dwell time of an analyte interaction or translocation event. Representative current traces recorded over 80 ms (c) for FPA (red) and FPA-P (blue) and over 1 s for FPA, FPA-P, and the mix (red, blue, and purple respectively; d–f). Representative scatter plots showing normalized blockade level, defined as (*I*_0_ – *I*_b_)/*I*_0_, against dwell time for each blockade (g–i), where faint dotted lines trace from the center of event populations to the *y* axis for blockade level, and arrows denote the different populations (FPA, red; FPA-P1, blue; FPA-P2, cyan). Representative histograms of blockade level as a function of the number of blockades (j–l), with insets showing magnified views of the low frequency events (k,l). Red, blue, and cyan lines are Gaussian fits to determine the most probable mean-normalized blockade levels were 0.63 ± 0.01 for FPA (red), 0.71 ± 0.02 for the first population of FPA-P (FPA-P1, blue) and 0.61 ± 0.01 for the second population (FPA-P2, cyan). The three populations identified in the mixture have the following mean-normalized blockade levels: 0.64 ± 0.01 (red), 0.70 ± 0.02 (blue), and 0.61 ± 0.02 (cyan). All experiments were conducted at *V* = +50 mV in 4 M KCl, 25 mM Tris HCl, pH 7.5 buffer. Data shown are from a single recording for each experiment, with the fitted values being the mean and standard deviation for three independent fits. *I*_0,FPA_ = 114.98 ± 1.67 pA; *I*_0,FPA-P_ = 116.66 ± 1.60 pA; *I*_0,mix_ = 118.25 ± 2.06 pA. *N*_FPA_ = 3339 events; *N*_FPA-P_ = 3259 events; *N*_mix_ = 2205 events.

Nanopore single-molecule electrical detection uses an applied electric field to drive individual molecules (analyte) into a single narrow pore (few nanometers in diameter) placed in an otherwise impermeable membrane between two compartments (Figure 2a). Passage of the analyte into the pore reduces the ionic current of the empty pore (*I*_0_; Figure 2b) to the blockade current (*I*_b_; Figure 2b) that is related to the size of the analyte relative to the pore diameter. The current is reduced for the duration that the analyte is within the pore (dwell time; Figure 2b), as determined by the combination of analyte charge, size, pore–analyte interactions, and the applied electrical forces. In the protein/peptide detection studies described above, only one parameter was measured with enough resolution to clearly identify and/or distinguish between the different peptide biomarkers: either the blockade level or the dwell time.

Here, the challenge is to directly show the detection and identification, at the single-molecule level, of a peptide biomarker and its derivatives, including the phosphorylated form (Figure 1), and to improve the reliability of identification by using two parameters: the dwell time, as well as the classical mean blockade level (Figure 2b). As a known family of biomarkers with a phosphorylated form that has physiological relevance, the fibrinopeptide A family serves as a medically important challenge for the proof-of-concept for this approach. We use a powerful sensor for biomolecule characterization of the aerolysin nanopore^53,54^ that was previously used for the discrimination of biomolecule size and sequence,^36,55,56^ chemical modifications,^49,50,57−60^ and conformation.^61,61−67^

In this study we show the proof-of-concept for characterization of a biomarker for several coagulation diseases using a wild-type aerolysin nanopore sensor. We first show that a single phosphorylation on FPA produces a unique electrical signal for this biomarker when compared with FPA without chemical modification. This signal comprises measurements for both the parameters of blockade level and dwell time for each of two observed FPA-P conformations. Using the same approach, we are also able to distinguish between the different FPA peptide derivatives of different lengths with these dual electrical parameters. Through the comparison of the signals produced by four of the FPA family derivatives using a wild-type (WT) aerolysin sensor, we demonstrate that each derivative has a unique electrical signature defined by two parameters: dwell time and current blockade level.

## Results and Discussion

### 1 Characterization and Discrimination of a Post-Translational Modification in a Coagulation Peptide Biomarker

To show that a WT aerolysin nanopore can discriminate between a peptide with- or without- a post-translational modification, such as the phosphorylation that occurs on fibrinopeptide A, we performed a series of independent experiments using FPA, FPA-P, and an equimolar mix of FPA and FPA-P (Figure 2). With this nanopore approach, two compartments (*cis* and *trans*) separated by a WT aerolysin nanopore inserted into a lipid bilayer are immersed in an electrolyte (4 M KCl, 25 mM Tris pH 7.5; Figure 2a). The application of a constant potential difference between the two electrodes of +50 mV in the absence of analyte enables the measurement of a stable ionic current (pA) flowing through the pore [open pore current *I*_0_, (Figure 2b)]. After adding the peptides into the *cis* compartment, entrance of FPA or FPA-P into the pore induces a detectable decrease (Δ*I*) of the open pore ionic current (*I*_0_), to the blockade current (*I*_b_) for the time the analyte occupies the pore (dwell time; Figure 2b–f). Scatter plots of the key parameters for each event, normalized Δ*I* (blockade level) against dwell time, show distinct event populations (Figure 2g,h), where normalized blockade current is defined as (*I*_0_ – *I*_b_)/*I*_0_. The first population observable for FPA and FPA-P corresponds to short events (<200 μs) with low blockade levels. These are characteristic of well-described bumping events, where molecules diffuse close to the pore entrance, thereby reducing the current for a short duration, before diffusing away. Translocation or interaction events are characterized by longer dwell times (>200 μs) and higher blockade levels, as indicated by the arrows. As in previous work,^50,56^ we observed only one population for FPA, characteristic of a peptide with uniform size and shape (Figure 2g). Interestingly, for FPA-P, we observed two discrete populations (FPA-P1 and FPA-P2; Figure 2h).

We plotted histograms of the normalized current blockade for all events to understand if these populations of events can be discriminated and if each peptide can be identified as a function of their relative blockade levels (Figure 2j,k). We measured one single peak localized at 0.63 ± 0.01 for FPA (red arrow), whereas we observed two peaks localized at 0.71 ± 0.02 for FPA-P1 (blue arrow) and 0.61 ± 0.01 for FPA-P2 (cyan arrow). Since blockade levels mainly depend on both the conformation and size of the analyte, the blockade level differences between FPA and FPA-P could be due to conformational changes induced by phosphorylation on Ser3. This is consistent with NMR studies that show clear signal changes when Ser3 is phosphorylated.^22,69^ Other work has shown that phosphorylation of short peptides can induce conformational changes.^70−74^ While nanopore sensing has been used to define the effect of phosphorylation on peptide conformation and has shown that this chemical modification leads to different current blockade signals,^45,47,49,50,58,59^ these studies have only observed a single population for the phosphorylated peptide.

Our work clearly shows the advantage of single molecule sensing in the detection of two discrete conformation states for FPA-P. While previous data suggests that many short peptides are conformationally flexible,^72^ phosphorylation is known to stabilize α-helices through interaction with the N-cap amine (when the phosphoserine is the third amino acid), as well as through the formation of salt bridges with lysine side chains four positions removed.^72,75^ NMR modeling of FPA-P in solution shows a stable helical turn in the C-terminal half of the peptide,^22^ while the seven N-terminal amino acids remained flexible. However, it is possible this ensemble data may not have the resolution to observe an alternative stable but less populated conformation, such as was observed in our data. The additional information provided by the detection of two discrete populations for FPA-P increases the probability of correct identification of this phosphorylated biomarker from mixed samples.

**Figure 3:**
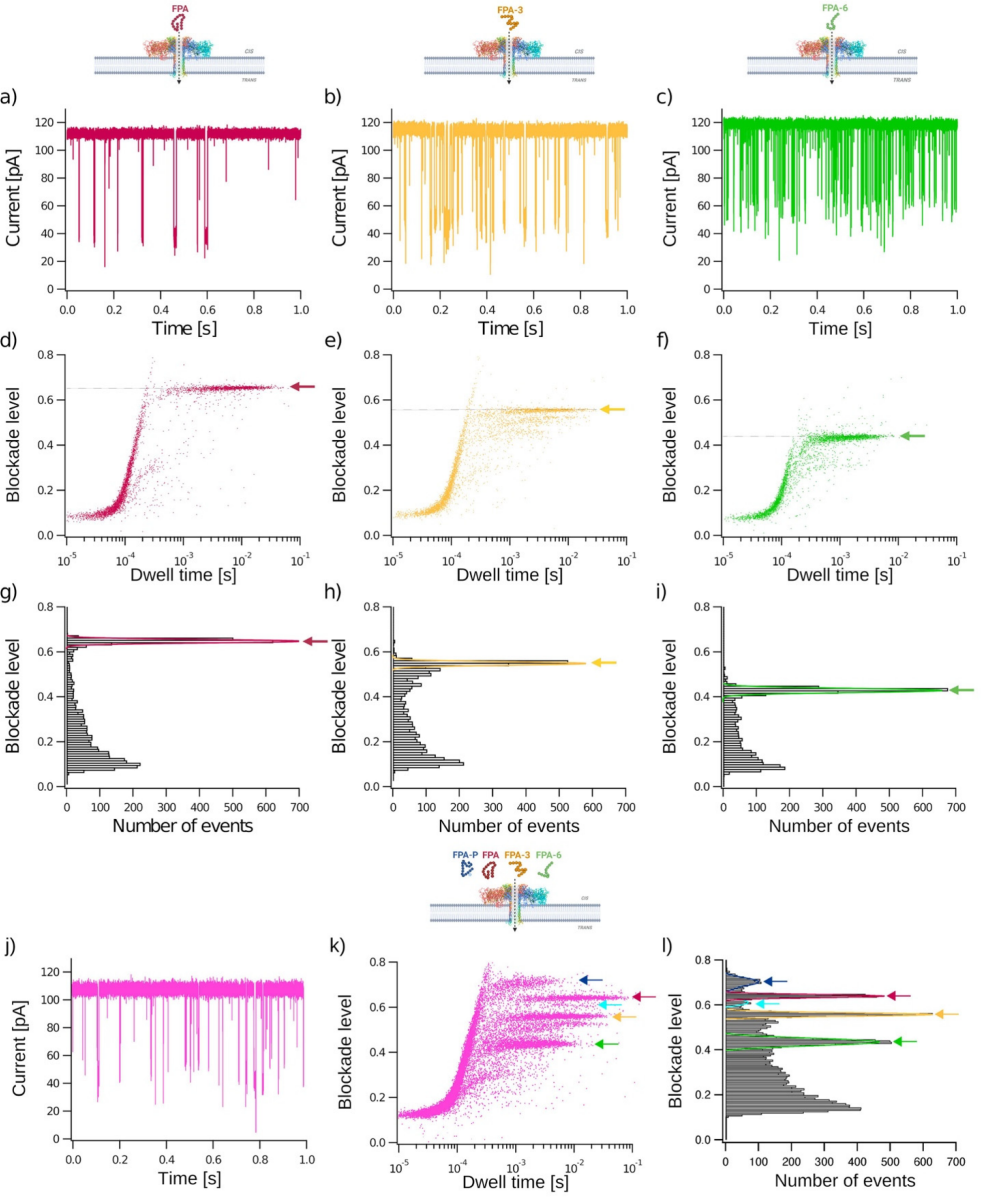
Characterization and discrimination of different fibrinopeptide A biomarkers. FPA (red), FPA-3 (yellow), and FPA-6 (green) peptides were analyzed separately at a 40 μM final concentration (a–i) and in a mixture of FPA, FPA-P, FPA-3, and FPA-6 (j–l). Representative current traces recorded through a wild-type aerolysin nanopore (PDB:5JZT) over 1 s for each peptide and the mixture (a–c,j). Bidimensional scatter plots showing blockade level against dwell time for each event (d–f,k). Normalized blockade current is (*I*_0_ – *I*_b_)/*I*_0_, where *I*_0_ is the open pore current, and *I*_b_ is the blockade current. Representative histograms of blockade level as a function of the number of events (g–i,l). Red, yellow and green lines are Gaussian fits to determine the most probable mean blockade level at 0.65 ± 0.01 for FPA, 0.55 ± 0.01 for FPA-3, and 0.43 ± 0.01 for FPA-6 (g–i). The five populations identified in the mixture of FPA (10 μM), FPA-P (20 μM), FPA-3 (10 μM), and FPA6 (2.5 μM) (l) have mean blockade levels of 0.70 ± 0.02 (FPA-P1, blue), 0.64 ± 0.01 (FPA, red), 0.62 ± 0.01 (FPA-P2, cyan), 0.56 ± 0.01 (FPA-3, yellow), and 0.44 ± 0.01 (FPA-6, green). All experiments were conducted at *V* = +50 mV, in 4 M KCl, 25 mM Tris pH 7.5 buffer. Data shown are from a single experiment, with the fitted values being the mean and standard deviation for three independent fits. *I*_0,FPA_= 112.02 ± 1.53 pA; *I*_0,FPA-3_ = 114.47 ± 1.87 pA; *I*_0,FPA-6_ = 118.78 ± 1.66 pA; *I*_0,mix_ = 106.82 ± 2.57 pA. *N*_FPA_ = 4474 events; *N*_FPA-3_ = 4230 events; *N*_FPA-6_ = 3742 events; *N*_mix_ = 19 435 events.

To this end, we tested this premise using an equimolar mix of FPA and FPA-P and found the same three event populations corresponding to the two peptides (Figure 2i,l). The mean blockade levels were found to be 0.64 ± 0.01 for FPA (red arrow), 0.70 ± 0.02 for FPA-P1 (blue arrow), and 0.61 ± 0.01 for FPA-P2 (cyan arrow), which was consistent with the individual experiments, as well as with the experiments using different relative concentrations of FPA and FPA-P (Figure S1 and Figure 3). This result shows that a WT aerolysin nanopore is powerful enough to detect a single post-translational modification, such as phosphorylation, as well as the landscape of induced conformation states.

### 2 Characterization and Discrimination of FPA Derivatives by Their Size

Since a series of FPA derivative peptides where the N-terminal amino acid was sequentially cleaved have been identified by mass spectrometry,^26−28^ we tested the efficacy of sensing these derivatives with our system (Figure 3). We first focused on the electrical characterization of FPA, FPA-3, and FPA-6 in independent experiments (Figure 3 a–i). The same short bumping events were found for each derivative. However, translocation or interaction events characterized by longer dwell times and higher current blockade levels are distinct for each derivative. The mean blockade levels are 0.65 ± 0.01, 0.55 ± 0.01, and 0.43 ± 0.01 for FPA, FPA-3, and FPA-6, respectively. It should be noted that the events with lower blockade levels and shorter dwell times for FPA-3 (Figure 3 e and k) are consistent with contamination by shorter impurities from synthesis, as has been previously observed.^56^ These results show that each peptide has a specific electrical signature using its average blockade level. Similar results were published where blockade levels decrease with peptide length.^36,56,76−79^

To confirm that each derivative can be independently discriminated from mixed samples, we performed experiments using a mixture containing each peptide (FPA, FPA-P, FPA-3, and FPA-6; Figure 3 j–l). As expected, the event blockades for each derivative on the raw current trace are not clearly discernible to the eye (Figure 3j). However, analysis of the parameters (blockade level and dwell time) for each event clearly shows distinct blockade levels for each peptide when presented in a scatter plot (Figure 3k). We identified five populations in the mix having mean blockade levels of 0.70 ± 0.02 (blue arrow), 0.64 ± 0.01 (red arrow), 0.61 ± 0.02 (cyan arrow), 0.56 ± 0.01 (yellow arrow), and 0.44 ± 0.01 (green arrow; Figure 3l), which is consistent with those observed in the independent experiments for each derivative of FPA, FPA-P, FPA-3, and FPA-6. These data demonstrate that we can discriminate between N-terminally cleaved derivatives and a post-translational modification of FPA from a mixture using the single parameter of blockade level.

### 3 Identification of FPA Derivatives Using Dual Electrical Parameters

**Figure 4:**
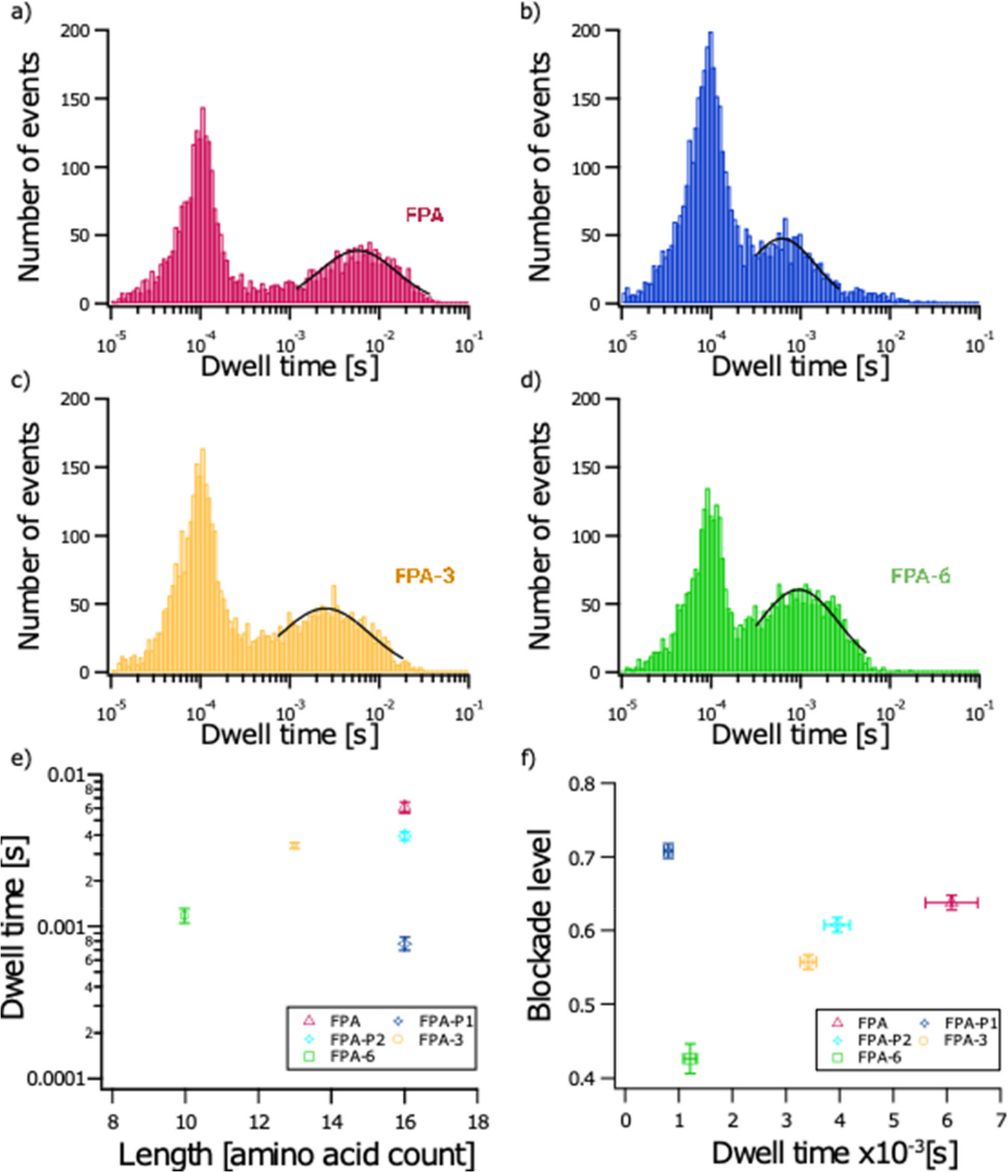
Event durations of different fibrinopeptide A biomarkers. Representative histograms of the number of events as a function of dwell time for FPA (red; a), FPA-P (blue; b), FPA-3 (yellow; d), and FPA-6 (green; d) peptides. Black lines are log-normal fits used to determine the most probable dwell times. Data shown are from a single recording for each experiment (independent experiments from those shown in Figures 2 and 3). Mean dwell time and standard deviations calculated from three independent fits to the data were: 5.74 ± 0.22 ms (FPA, red); 0.62 ± 0.04 ms (FPA-P1, blue); 4.41 ms ± 0.33 ms (FPA-P2, cyan); 2.50 ± 0.12 ms (FPA-3, yellow); and 0.95 ± 0.04 ms (FPA-6, green). *N*_FPA_ = 3339 events; *N*_FPA-P_ = 4309 events; *N*_FPA-3_ = 4230 events; *N*_FPA-6_ = 3742 events (a–d). Plot showing mean and standard deviation of dwell time (calculated from the fits to data from three independent experiments) as a function of peptide length: 6.09 ± 0.49 ms (FPA, red); 0.77 ± 0.08 ms (FPA-P1, blue); 3.94 ± 0.24 ms (FPA-P2, cyan); 3.40 ± 0.15 ms (FPA-3, yellow); 1.18 ± 0.13 ms (FPA-6, green) (e). Plot showing mean and standard deviations for dwell time as a function of blockade level for each peptide calculated from the fits to data from three independent experiments: 0.64 ± 0.01 (FPA, red); 0.71 ± 0.01 (FPA-P1, blue); 0.61 ± 0.01 (FPA-P2, cyan); 0.56 ± 0.01 (FPA-3, yellow); and 0.43 ± 0.02 (FPA-6, green) (f).

To the best of our knowledge, none of the previously published studies have shown that wild-type aerolysin could discriminate peptide biomarkers, as opposed to model peptide sequences, with the combination of their most probable dwell times and their characteristic blockade level. In fact, by increasing the number of defined electrical parameters that can be used to identify a molecule, one can expect that the probability of correct identification would also increase. To prove so, in this present study, we first extracted the characteristic dwell times for each population of events found for FPA, FPA-P, FPA-3, and FPA-6. We found the following average most probable dwell times: 5.74 ± 0.22 ms for FPA; 0.62 ± 0.04 ms for FPA-P1; 4.41 ms ± 0.33 ms for FPA-P2; 2.50 ± 0.12 ms for FPA-3; and 0.95 ± 0.04 ms for FPA-6 (Figure 4a–d). These data show that each peptide can be characterized by its unique average dwell time, even for FPA and both conformations of FPA-P, which have the same size (16 amino acids) but only differ by a single phosphorylation on Ser3 or in conformation (Figure 4e).

Finally, we plotted the average blockade level of each event population described above as a function of their average most probable dwell time (Figure 4f). Using this bidimensional plot, we show that each population of events can be clearly characterized and identified by these two physical parameters.

## Conclusion

In summary, we have demonstrated that WT aerolysin has the resolving power to detect and discriminate between phosphorylated and unmodified FPA, as well as between different cleavage derivatives of FPA. Interestingly, for the first time, two populations of events were observed for the phosphorylated FPA. Not only are these events resolvable with the classical parameter of blockade level, but each peptide also has different profiles for the dwell time parameter. The combination of these two parameters provides a unique signature that can be used to detect and identify these peptides from mixed samples. Of particular interest is the unique signature observed for FPA-P that essentially comprises four parameters, that is, dwell time and blockade level for two different conformations, which provides additional parameters that increase the potential for identification of this phosphorylated fibrinopeptide biomarker.

A key challenge to developing a POC nanopore assay requires increasing the nanopore interaction frequency for low concentration analytes since the physiologically relevant concentration of many biomarkers is in the picomolar to low nanomolar range. This could be achieved by a 2-fold approach: creating aerolysin variants that increase the electro-osmotic force that drives the analyte into the pore,^80,81^ and development of a robust and precise hybrid nanopore system^82−85^ to allow the use of higher driving forces (e.g., voltage and pressure) that will increase the event frequency for low concentration analytes.

Future discrimination of individual biomarkers from complex biological samples will require the characterization of additional parameters that could be used for analyte identification. Such parameters could be determined from blockade current fluctuation, including analysis of the standard deviation of the event blockade current^86,87^ or definition of internal steps.^88−92^ The combination of parameters, including those from alternative conformations such as those observed for FPA-P, could be used to classify individual or combinations of events with clustering algorithms such as fuzzy-c means, hidden Markov models, or density-based methods.^91−93^ The application of machine learning can further exploit such classification.^32,94−97^ While these in-depth data analysis approaches are well developed for DNA sequencing,^98^ their application to the identification of single analytes is still in development. In the future, these data processing and analysis techniques will facilitate the specific identification of a signature parameter combination for individual biomarkers.

In summary, we here provide proof-of-concept that aerolysin nanopore sensing can discriminate between phosphorylated and unphosphorylated FPA and its shorter derivatives. While many challenges remain to be overcome, this work represents a step in the process of developing a rapid, direct POC test for this medically important biomarker.

## Methods

### Aerolysin Production and Activation

WT aerolysin was produced by Dreampore S.A.S. (Cergy, France). Briefly, protein was expressed in BL21 Rosetta2 cells, as previously described,^99^ and purified by nickel affinity and desalting chromatography (Cytiva, Malborough MA, USA) in standard buffers containing 350 mM NaCl buffer, concentrated to ∼1 μM and stored at 4 °C until use. Aerolysin was activated with trypsin immobilized on agarose beads (Thermo Scientific, Waltham MA, USA) for 15 min at 20 °C.

### Peptides

Fibrinopeptides FPA (Nter-ADSGEGDFLAEGGGVR-Cter), FPA-P (ADS*GEGDFLAEGGGVR), FPA3 (GEGDFLAEGGGVR), and FPA6 (DFLAEGGGVR) were synthesized and purified (HPLC) by Proteogenix (Schiltigheim, France) and resuspended to 1 mM in 25 mM Tris pH 7.5.

### Nanopore Setup

A vertical planar lipid bilayer setup (Warner Instruments, Hamden CT, USA) was used to perform the nanopore experiments, as previously described.^63^ A planar lipid bilayer was formed from diphytanoylphosphatidylcholine (DphPC), dissolved to 10 mg/mL in decane, to create a membrane separating two compartments containing 1 mL each of 25 mM Tris pH 7.4 and 4 M KCl. An approximately 1 nM final concentration of activated aerolysin monomers was used to achieve nanopore insertion. Ag/AgCl electrodes were used to apply the voltage and measure the current signal. Peptide sensing experiments were conducted with 40 μM of one peptide with an applied voltage of +50 mV. Peptide mix discrimination experiments were conducted with different concentrations of each peptide with an applied voltage of +50 mV.

### Data Acquisition

The ionic current through a single nanopore was measured using an Axopatch 200B amplifier. Data were filtered at 5 kHz and recorded at 250 kHz intervals (4 μs sampling time) using a DigiData 1440A digitizer and Clampex software (Axon Instruments, Union City, CA, USA). Data were processed and analyzed such that event detection was defined by a threshold statistically determined from the average baseline (open pore) current (*I*_0_) and its standard deviation (δ) using the calculation *I*_0_ – 5δ.^100^ Low-frequency events were analyzed from data concatenated from multiple recordings. Parameters for dwell time and average blockade current were extracted for each event, with the latter further used to calculate the normalized blockade level using the relationship (*I*_0_ – *I*_b_)/*I*_b_. Histograms of the dwell time and blockade level were fit to log-normal and Gaussian distributions, respectively, to determine the most probable values for the dwell time and normalized blockade current, with the mean and standard deviation for each parameter determined from three independent fits to the data. Plots in Figure 4e,f show the mean and standard deviations of fits to groups of events selected by defining a box covering the distribution for their normalized blockade level and dwell time from three independent experiments. All data processing, fitting, and plot generation were conducted using Igor Pro Software (Wavemetrics Inc., Portland, OR, USA).

## Supporting Information Available

Additional data from experiments using different concentrations of FPA and FPA-P (PDF)
