# Supplementary material for: Nanopore Discrimination of Coagulation Biomarker Derivatives and Characterization of a Post-Translational Modification
Source: ACS Cent Sci. 2023 Feb 3;9(2):228–38. doi: 10.1021/acscentsci.2c01256 (PMC9951287; doi:10.1021/acscentsci.2c01256)
Supplement: Supplementary file 1 — oc2c01256_si_001.pdf [file oc2c01256_si_001.pdf]

Supporting Information for

Nanopore discrimination of coagulation biomarker derivatives and characterization of a post-translational modification

Aïcha Stierlen[1], Sandra J. Greive[2], Laurent Bacri[3], Philippe Manivet[4,5], Benjamin Cressiot[1*], Juan Pelta[1,3*]

1 LAMBE, CNRS, CY Cergy Paris Université, 95000, Cergy, France

2 DreamPore S.A.S., 33 Boulevard du Port 95000, Cergy, France

3 LAMBE, CNRS, Univ Evry, Université Paris-Saclay, 91025, Evry-Courcouronnes, France

4 Centre de Ressources Biologiques Biobank Lariboisière (BB-0033-00064), DMU BioGem, AP-HP, France

5 Université Paris Cité, Inserm, NeuroDiderot, F-75019, Paris, France

* Corresponding authors: benjamin.cressiot@cyu.fr; juan.pelta@univ-evry.fr

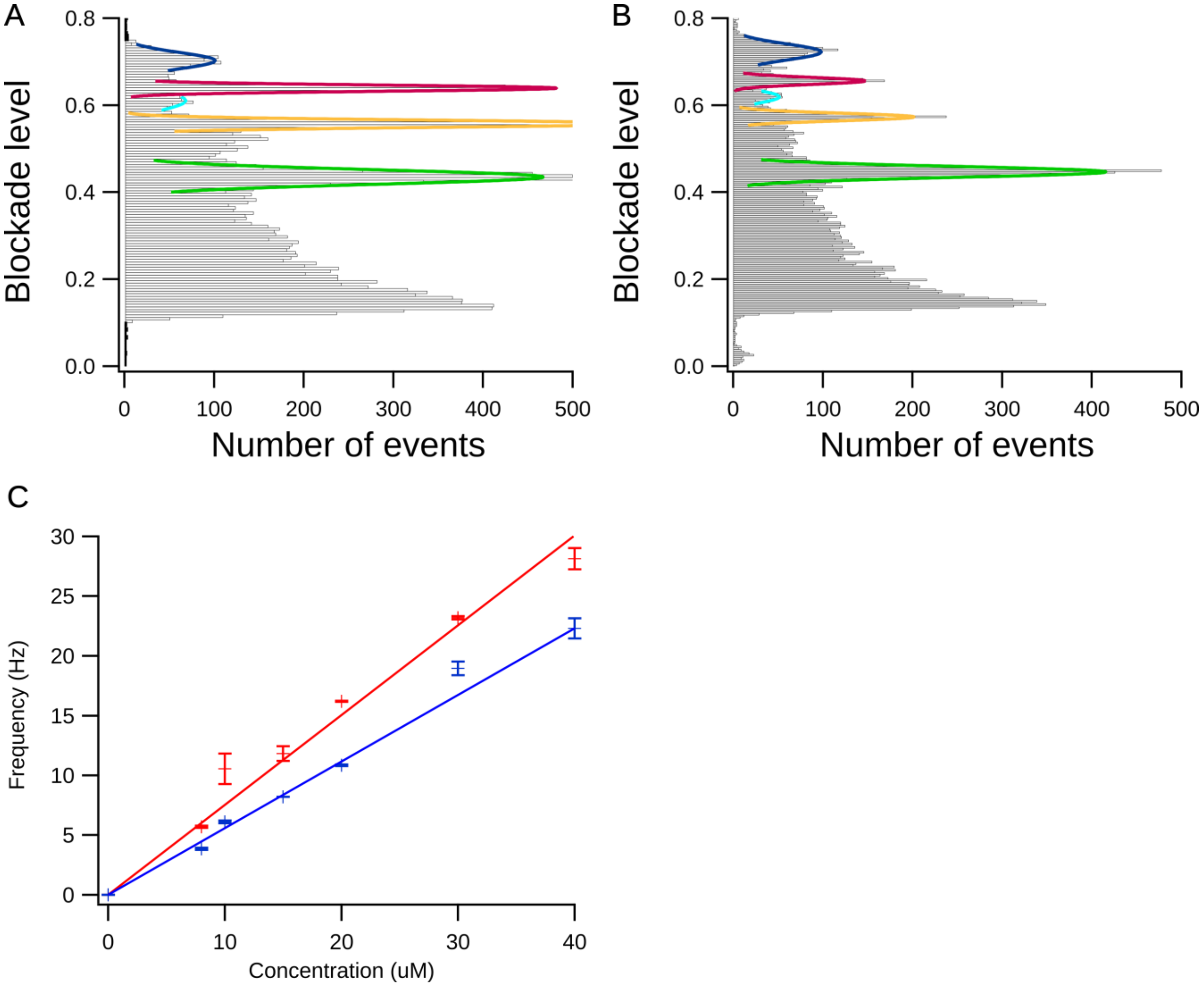

Figure S1: Detection of FPA and FPA-P at different concentrations. A and B) Histograms of the normalized blockade level against number of events for mixtures of FPA (red), FPA-P (populations: P1, blue; and P2, cyan), FPA-6 (yellow) and FPA-3 (green). Colored lines are fits of Gaussian functions to the data defining the most probable blockade level. A) Histogram of an equimolar mixture at 10 µM (FPA:FPA-P 1:1) where the average and standard deviation of three fits to the data from 6221 events determined blockade levels for FPA 0.65 ± 0.01 (red), FPA-P1 0.71 ± 0.01 (blue), FPA-P2 0.62 ± 0.01 (cyan), FPA-3 0.57 ± 0.01 (yellow) and FPA-6 0.44 ± 0.01 (green). B) Histogram for an equimolar mix of each peptide at 2.5 µM except for FPA-P which was at 20 µM (FPA:FPA-P 1:8). The average and standard deviation for the most probable blockade level, from three fits to the data from 20687 events, determined blockade levels of FPA 0.0.66 ± 0.01 (red), FPA-P1 0.72 ± 0.02 (blue), FPA-P2 0.62 ± 0.01 (cyan), FPA-3 0.57 ± 0.01 (yellow) and FPA-6 0.45 ± 0.01 (green). C) Plot of frequency vs concentration for FPA (red) and FPA-P (blue). Lines are linear fits to the data to generate a concentration curve.
